# The Hitchhiker Guide to CD4^+^ T-Cell Depletion in Lentiviral Infection. A Critical Review of the Dynamics of the CD4^+^ T Cells in SIV and HIV Infection

**DOI:** 10.3389/fimmu.2021.695674

**Published:** 2021-07-21

**Authors:** Quentin Le Hingrat, Irini Sereti, Alan L. Landay, Ivona Pandrea, Cristian Apetrei

**Affiliations:** ^1^ Division of Infectious Diseases, DOM, School of Medicine, University of Pittsburgh, Pittsburgh, PA, United States; ^2^ HIV Pathogenesis Section, Laboratory of Immunoregulation, National Institute of Allergy and Infectious Diseases, National Institutes of Health, Bethesda, MD, United States; ^3^ Department of Internal Medicine, Rush University Medical Center, Chicago, IL, United States; ^4^ Department of Pathology, School of Medicine, University of Pittsburgh, Pittsburgh, PA, United States; ^5^ Department of Infectious Diseases and Immunology, Graduate School of Public Health, University of Pittsburgh, Pittsburgh, PA, United States

**Keywords:** human immunodeficiency virus, simian immunodeficiency virus (SIV), AIDS, microbial translocation, immune activation (IA), inflammation, CD4^+^ T cells

## Abstract

CD4^+^ T-cell depletion is pathognomonic for AIDS in both HIV and simian immunodeficiency virus (SIV) infections. It occurs early, is massive at mucosal sites, and is not entirely reverted by antiretroviral therapy (ART), particularly if initiated when T-cell functions are compromised. HIV/SIV infect and kill activated CCR5-expressing memory and effector CD4^+^ T-cells from the intestinal lamina propria. Acute CD4^+^ T-cell depletion is substantial in progressive, nonprogressive and controlled infections. Clinical outcome is predicted by the mucosal CD4^+^ T-cell recovery during chronic infection, with no recovery occurring in rapid progressors, and partial, transient recovery, the degree of which depends on the virus control, in normal and long-term progressors. The nonprogressive infection of African nonhuman primate SIV hosts is characterized by partial mucosal CD4^+^ T-cell restoration, despite high viral replication. Complete, albeit very slow, recovery of mucosal CD4+ T-cells occurs in controllers. Early ART does not prevent acute mucosal CD4^+^ T-cell depletion, yet it greatly improves their restoration, sometimes to preinfection levels. Comparative studies of the different models of SIV infection support a critical role of immune activation/inflammation (IA/INFL), in addition to viral replication, in CD4^+^ T-cell depletion, with immune restoration occurring only when these parameters are kept at bay. CD4^+^ T-cell depletion is persistent, and the recovery is very slow, even when both the virus and IA/INFL are completely controlled. Nevertheless, partial mucosal CD4^+^ T-cell recovery is sufficient for a healthy life in natural hosts. Cell death and loss of CD4^+^ T-cell subsets critical for gut health contribute to mucosal inflammation and enteropathy, which weaken the mucosal barrier, leading to microbial translocation, a major driver of IA/INFL. In turn, IA/INFL trigger CD4^+^ T-cells to become either viral targets or apoptotic, fueling their loss. CD4^+^ T-cell depletion also drives opportunistic infections, cancers, and comorbidities. It is thus critical to preserve CD4^+^ T cells (through early ART) during HIV/SIV infection. Even in early-treated subjects, residual IA/INFL can persist, preventing/delaying CD4^+^ T-cell restoration. New therapeutic strategies limiting mucosal pathology, microbial translocation and IA/INFL, to improve CD4^+^ T-cell recovery and the overall HIV prognosis are needed, and SIV models are extensively used to this goal.

## Introduction

Even before HIV was formally established as the cause of AIDS, CD4^+^ T-cell depletion was identified as a key feature of HIV infection. Indeed, lymphocytopenia was among the first biological findings described in the early days of the AIDS pandemic (1). Lymphocytopenia was notably due to a depletion of CD4^+^ T cells and, in addition to their decrease in absolute number and percentage of total T cells, residual CD4^+^ T cells were dysfunctional in AIDS patients (1). Later, CD4 was identified as HIV/SIV receptor (2, 3), and CD4^+^ T cell counts in peripheral blood were reported to predict the risk of progression to AIDS (4).

However, the notions of CD4^+^ T-cell depletion and restoration encompass processes that are vastly different for different CD4^+^ T-cell subsets and according to their tissue location (5–8). As longitudinal studies cannot access and sample all body compartments, reasonable knowledge on CD4^+^ T-cell dynamics during HIV infection was obtained from descriptive studies of cohorts of HIV-1 infected patients and experimental studies of simian immunodeficiency virus (SIV) infection in nonhuman primates (NHP). Depending on whether or not an NHP is a natural host of SIV (9), the infection will be nonpathogenic or pathogenic, respectively, and CD4^+^ T-cell dynamics will also vary accordingly.

Here, we will review the general features of the depletion of the different CD4^+^ T-cell subsets and their restoration during pathogenic and nonpathogenic HIV/SIV infections, as well as the consequences of CD4^+^ T-cell depletion, and the potential approaches that could help reverse CD4^+^ T-cell depletion and prevent its deleterious consequences.

## CD4^+^ T-Cell Subsets

Different CD4^+^ T-cell subsets are defined, according to their differentiation status or phenotype. CD4^+^ T cells from humans and NHP are classified as naïve (CD45RA^+^ CCR7^+^ CD28^+^ CD95^neg^) or memory T cells (CD45RO^+^ CD95^+^) (10, 11). Memory T cells are subdivided into stem cell memory (Tscm; CD45RA^+^ CCR7^+^ CD28^+^ CD95^+^) (12, 13), central memory (Tcm; CD45RA^neg^ CCR7^+^ CD62L^+^) (11), transitional memory (Ttm; CD45RA^neg^ CCR7^neg^ CD95^+^ CD62L^+^), effector memory (Tem; CD45RA^neg^ CCR7^neg^ CD95^+^ CD28^neg^ CD62L^neg^) (14), terminal effector (Tte; CD45RA^+^ CCR7^neg^ CD95^+^ CD28^neg^ CD62L^neg^) (14) and resident memory (Trm; CD45RA^neg^ CCR7^neg^ CD69^+^ ± CD103^+^) (15) T cells. Meanwhile, based on their functional status, CD4^+^ T cells can be classified as Th1 (IFN-γ producing; transcription factor: T-bet), Th2 (IL-4 producing; GATA3), Th17 (IL-17 producing; RORγt), regulatory T cells (Tregs) (suppressive function; FoxP3) and follicular helper T cells (Tfh) (IL-21 producing; Bcl6).

Additional phenotypic markers can be used to differentiate CD4^+^ T cells, notably CCR5, the main coreceptor of HIV/SIV (16–18), and markers of cell proliferation (Ki-67, BrdU), activation (HLA-DR, CD38, CD69), exhaustion (PD-1, CTLA-4, Tim-3) or senescence (CD57, KLRG-1) (19).

Finally, CD4^+^ T cells can also be subdivided according to their metabolic status, with memory cells tending to have higher metabolic activities, notably glycolysis and oxidative phosphorylation (20).

The multiple CD4^+^ T cell populations defined using any of these features are differentially infected, depleted, and restored. Note that during the course of the HIV/SIV infection other immune cells (CD4^+^CD8^+^ T cells, γδTCR T cells, innate lymphoid cells type 2 and 3, circulating dendritic cells) (21–26) can be depleted and restored, but their dynamics will not be detailed in this review.

## CD4^+^ T-Cell Depletion in Pathogenic HIV/SIV Infections

### Circulation and Lymph Nodes

When CD4^+^ T-cell depletion was first investigated in people with HIV (PWH), research focused on CD4^+^ T cells dynamics in blood and superficial LNs, notably tonsils, as they were more accessible. Circulating CD4^+^ T cells are moderately depleted during acute HIV or SIV infection (21, 27, 28). A slight increase in the CD4^+^ T cell counts occurs as a consequence of the postacute partial control of viral replication and establishment of the steady-state set-points (27, 29). During chronic HIV/SIV infection, a slow and continuous decline of circulating CD4^+^ T cells is observed, eventually leading to severe lymphopenia, rendering persons living with HIV susceptible to opportunistic infections and eventually leading to AIDS (30). Annual rate of CD4^+^ T cell decline in circulation in untreated patients is correlated to plasma viral loads, and was found to be higher in persons living with HIV-1 than with HIV-2 (-15.9% *vs*. -4.1% per year, respectively), due to major differences in the levels of viral replication between these two infections (31). Meanwhile, in the superficial and mesenteric lymph nodes, CD4^+^ T-cell depletion is minimal during acute HIV or SIV infection (32–35). However, during the very advanced stages of HIV/SIV infection, CD4^+^ T-cell depletion may occur even in lymph nodes and is associated with lymphadenopathy and fibrosis (34, 36, 37).

With the discovery that HIV-1, HIV-2, and most of the SIV use the chemokine receptor CCR5 as a coreceptor (16–18), and therefore preferentially infect memory T cells (38, 39), numerous studies then aimed at describing the dynamics of the different CD4^+^ T-cell subsets in PWH and SIV-infected NHPs. In young, uninfected humans, most CD4^+^ T cells in peripheral circulation and the lymph nodes are naïve, while the fraction of naïve CD4^+^ T cells declines in older individuals (40). Naïve T cells also represent the majority of CD4^+^ T cells in blood and lymph nodes of young (less than 4 years) rhesus macaques (RMs), the animal model of reference for HIV infection (11, 30). In Indian RMs, it has been estimated that, on average, less than 15% of CD4^+^ T cells from circulation and the lymph nodes express CCR5 (30, 32, 41), while over 75% of them express CXCR4. In both humans and macaques, the vast majority of CCR5^+^ CD4^+^ T cells are memory T cells (30, 42, 43). As a result, the majority of circulating CD4^+^ T cells are not direct targets for HIV and SIV, as emphasized by the low fraction of circulating CD4^+^ T cells that are HIV-infected (44). This could explain why the loss of peripheral CD4^+^ T cells is limited to 50-60% in most patients during acute HIV infection, with a median nadir of CD4^+^ T cells ranging between 340 and 510/mm^3^ (27, 45, 46). Similar decline in circulating CD4^+^ T cells is also observed in SIV-infected NHP during acute pathogenic (21), non-pathogenic (47) and controlled SIV infections (48). In addition to this total CD4^+^ T-cell depletion, a preferential depletion of the memory CD4^+^ T cells, especially those expressing CCR5, can be seen as early as 7-14 days postinfection (dpi) in the lymph nodes and periphery, whereas naïve T cells are preserved (6, 28, 32, 49).

Once the strategies for the detailed characterization of the different memory CD4^+^ T-cell subsets, notably Tcm, Ttm, Tem, became available, studies were performed on sorted CD4^+^ T-cell subsets to assess the frequency of infection, in addition to monitoring the evolution of each of those subsets throughout HIV/SIV infection. These studies established that, in blood, Tcms represent the major cellular reservoir in HIV-1 infected individuals (50), while Ttms and/or Tems form the bulk of the reservoir in HIV-1 long term nonprogressors, SIVsmm-infected sooty mangabeys (a prototypic nonpathogenic infection) and HIV-2-infected individuals (51–53). In pathogenic hosts of SIV, the frequency of HIV/SIV infected cells is also high in the recently described Tscm subset, which expresses high levels of CCR5 (5, 54). In patients with progressive infection, as a result of a prolonged and continuous depletion of the target Tcm and Tem cells, due to cell death and reduced proliferation of Tcm, the majority of the remaining CD4^+^ T cells are naïve (5, 6).

Dynamics of the CD4^+^ T cells with specific functions have also been probed. Both HIV and SIV preferentially infect Th1 and Th17 cells (55). As a result, HIV and SIV infections are characterized by a switch from Th1 to Th2 phenotype (56), and a significant depletion of Th17 cells is observed among circulating lymphocytes throughout the infection (7). HIV-1 can also infect Tregs (57), and, although reduced (7, 58–60), stable, and increased (61, 62) absolute circulating Treg counts have all been reported during chronic infections, a decreased Th17/Treg ratio is commonly observed in pathogenic infections and was linked to immune activation and disease progression (7, 62). Furthermore, a selective depletion of circulating CD4^+^ T cells with gut homing potential (i.e., expressing the α4β7 integrin) preferentially occurs in untreated PWH (63) and in SIV-infected RMs in which those cells are selectively infected in the first days of infection (8). Most CD4^+^ T cells expressing α4β7 integrin are Tcm with a Th17 phenotype, and their dynamics in blood reflects the evolution of intestinal CD4^+^ T cells in jejunum (8, 64).

A subset of CD4^+^ T cells T follicular helper (Tfh), identified based on the expression of the surface markers CXCR5^+^ PD-1^high^, and preferentially found in B follicles in lymph nodes and spleen, can also be infected by HIV/SIV and is slightly depleted during acute infection, before accumulating during chronic infection (65–68). This chronic accumulation of Tfh might be due to a lack of regulation by the local Tregs, the follicular regulatory T cells (Tfr), as suggested by the decreased Tfr/Tfh ratio (69). Tfh cells are depleted during the AIDS stage (65). Signals provided by Tfh cells are crucial for the development of memory B cells, and the expansion of Tfh cells has been associated with B cell dysregulation, notably a reduced number of antigen-specific memory B cells, increased germinal center B cells, hypergammaglobulinemia, and lower Env-specific antibody titers (67, 70).

Finally, *in vivo*, CD4^+^ T cells can be also selectively infected according to their metabolic status (71). HIV-1 tends to infect CD4^+^ T cells with high oxidative phosphorylation and glycolysis, two metabolic activities more frequently enhanced in memory CD4^+^ T cells (71). The dynamics of CD4^+^ T cells during HIV/SIV infection according to their metabolic activities remain to be determined.

### Gastrointestinal (GI) Tract

While the circulating lymphocytes only account for 2 to 5% of the total lymphocytes, intestinal lymphocytes represent a tremendous fraction of total lymphocytes (over 60%) in both humans and NHPs. In the GI tract, lymphocytes exist in three major forms: (i) intraepithelial lymphocytes (IEL), (ii) lamina propria lymphocytes (LPL), and iii) lymphocytes organized in lymphoid formations (i.e., the Peyer’s patches and the solitary lymphoid follicles). There are strong similarities between human and NHPs’ gut-associated lymphoid tissue (GALT) regarding the distribution of the immune cells, with the CD4/CD8 ratios being about 1:2 and 1:1 in IEL and LPL, respectively (72).

In the years following the identification of AIDS, prompted partly by the high frequency of enteropathies in PWH (73), histological studies identified a loss of CD4^+^ T cells in gastrointestinal biopsies of PWH (74–79). Lim and colleagues also proved that, similar to circulation, memory CD4^+^ T cells were the preferentially depleted cell subset in the gut during HIV infection (80). However, results were sometimes contradictory, some studies reported that CD4^+^ T-cell depletion only affected the LPLs, while others described CD4^+^ T-cell depletion as impacting both LPLs and IELs (77, 81). Furthermore, almost all intestinal biopsies were obtained from clinically indicated procedures in patients presenting with AIDS or late-stage disease, limiting the insights on the dynamics of CD4^+^ T-cell depletion at this site (75).

**Figure 1:**
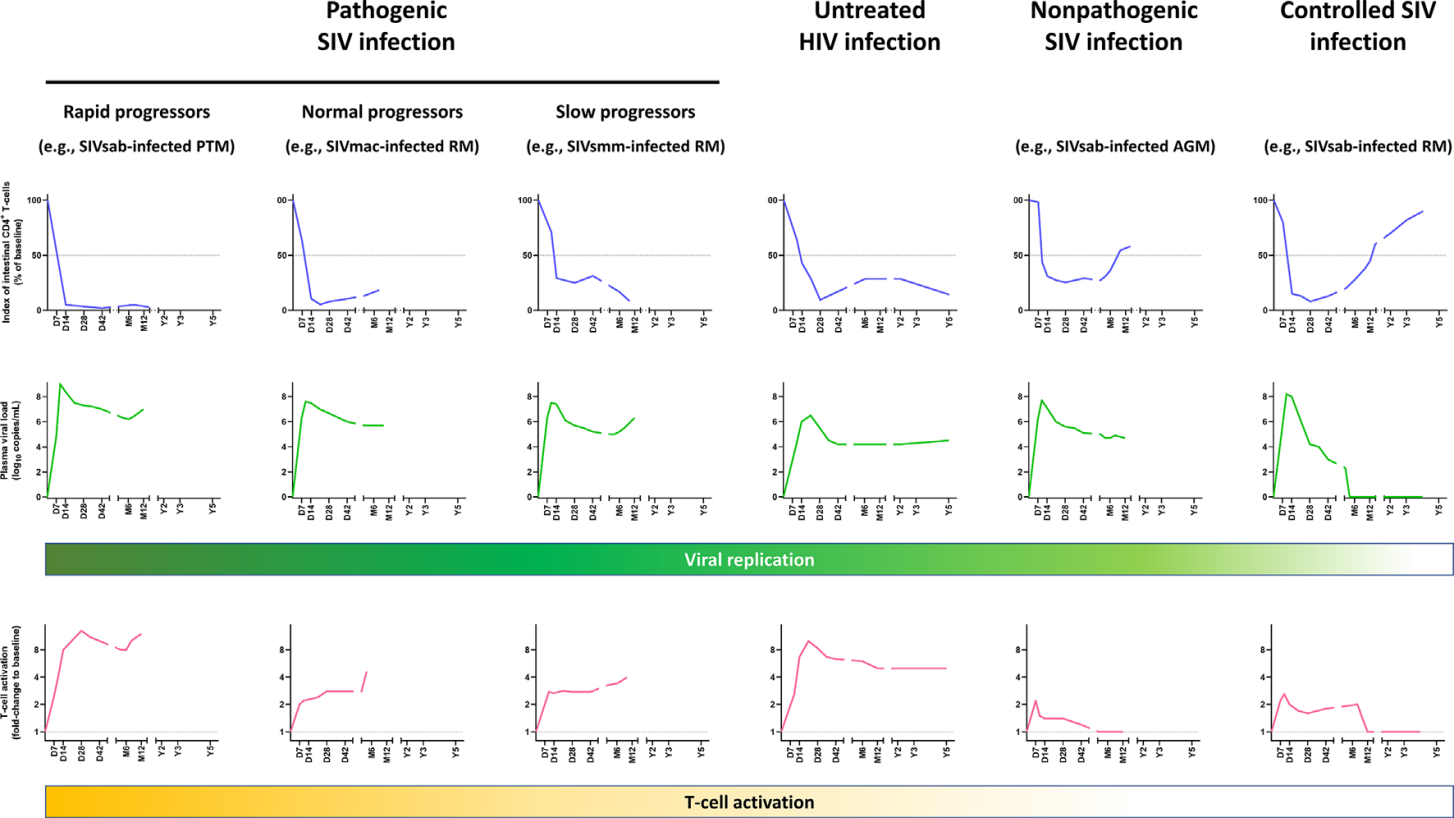
Comparative dynamics of intestinal CD4^+^ T-cell depletion, plasma viral loads and immune activation/inflammation in untreated HIV and SIV infections. Schematic representation of intestinal CD4^+^ T-cell depletion, viral replication and immune activation is inferred from data reported in references (21, 33, 48, 84–91). Longitudinal data are presented, with the X axis representing days (D), months (M) and years (Y) postinfection. The Y axis illustrates the magnitude of lamina propria CD4^+^ T-cell depletion (upper panels, blue), viral replication (middle panels, green), and the levels of T-cell activation (lower panels, yellow). Intestinal CD4^+^ T-cell depletion is illustrated as the index of lamina propria CD4^+^ T cells (i.e., percentage of CD4^+^ T cell fraction within the CD3^+^ T cell population, divided by this percentage at baseline). Viral replication is represented as plasma viral loads. From the plethora of biomarkers of immune activation/inflammation, we selected the fold-change of HLA-DR^+^ CD8^+^ T cells in SIV-infected NHPs, compared to the baseline preinfection levels, except for persons living with HIV, for which the fold-changes of HLA-DR^+^ CD38^+^ CD8^+^ T cells were used. Note that in other primate models of rapid progressors, T-cell activation might be more blunted (92). AGM, African green monkeys; PTM, Pigtailed macaques; RM, Rhesus macaques.

It was only a decade later that, due to research performed in SIV-infected RMs, the early dynamics of CD4^+^ T-cell depletion in GI tissues were described (21, 33, 82). These studies reported that a massive CD4^+^ T-cell depletion occurs as early as 7 dpi in SIVmac-infected RMs, leading to more than 90% of intestinal CD4^+^ T cells being lost at 14-21 dpi (21, 33, 83) (**Figure 1**). SIV-infected cells can be detected within 7 dpi in the gut (21, 33, 93). The peak of viral replication in the gut occurs approximately 10 dpi and the vast majority of the SIV-infected cells during the acute infection are found within the lamina propria, the remaining infected cells being mainly detected in organized lymphoid tissues and macrophages (33, 77, 94). CD4^+^ T-cell depletion occurs earlier in the jejunum than in the ileum and colon, and affects mostly LPL (33), probably because most of the lymphocytes in jejunum are found within the lamina propria, while organized lymphoid tissues are more common to the ileum and colon. The first studies reporting this massive and rapid CD4^+^ T-cell depletion in the gut were performed on animals intravenously inoculated, but later the same pathogenic features were confirmed in studies in which animals were infected either intrarectally or intravaginally (83, 94).

Phenotypic analyses of the intestinal CD4^+^ T cells enabled further characterization of the CD4^+^ T-cell subsets that were preferentially depleted in the gut. Contrary to circulating lymphocytes, most of the CD4^+^ T LPL have an activated (HLA-DR^+^ CD25^+^) and memory (CD45RA^neg^ CD95^+^) phenotype (49, 95). Activated, CCR5-expressing memory CD4^+^ T cells, which are the preferential targets of HIV and SIV, are frequent in the gut (30, 49, 96). The predominance of this activated, memory CD4^+^ T cell phenotype at the mucosal sites is common to both humans and macaques, being observed even in the newborns (95), and likely occurs as a consequence of exposure to antigens *in utero*. Interestingly, mucosal CD4^+^ T cells of humans and NHPs that are not natural hosts of SIV express higher levels of CCR5 compared to African natural host species (30, 41, 47, 84). At 14 dpi, virtually all memory CCR5^+^ CD4^+^ T cells are lost in the lamina propria and effector sites of the gut of SIV-infected RMs, with the spared intestinal CCR5^+^CD4^+^ T cells being naïve T cells (30). This swift depletion of CD4^+^ T cells has been confirmed to occur as well during acute HIV-1 infections (35, 97–99), and intestinal CD4^+^ T-cell depletion persists through the course of the disease in untreated PWH (35) (**Figure 1**).

As described for the circulating CD4^+^ T cells, several experiments have shown that the initial CD4^+^ T-cell depletion in the gut is driven by the coreceptor usage of the virus. NHP infection with X4-tropic viruses resulted in a rapid, profound depletion of the naïve CXCR4^+^ CD4^+^ T cells from the circulation and the lymph nodes, instead of the canonical depletion of memory CD4^+^ T cells in the GALT, typical of the R5-tropic viruses (100–102).

Furthermore, mucosal CD4^+^ T-cell depletion in the gut directly affects cell subsets that are involved in the maintenance of the mucosal barrier. As such, HIV/SIV infection can disrupt the production of IL-17 and IL-22, two cytokines that are essential for maintaining the tight epithelial barrier of the GI tract and gut integrity, by selectively depleting the lymphocytes producing those cytokines. Indeed, while Th1 and Th17 cell subsets are equally depleted in peripheral blood and they did not differ in frequency of infected cells, Th17 memory CD4^+^ T cells are selectively depleted from the lamina propria and effector sites of the GI tract of PWH and SIV-infected RMs, as early as 2 weeks postinfection (55, 62, 85, 103). lL-22 producing lymphocytes are also depleted during SIV (104, 105) and HIV infections (85). Other IL-17 and/or IL-22 producing cells exist in the GI tract, but for most of them a preferential depletion and/or a reduced IL-17 production has been reported during SIV infection (25, 105–107). Moreover, IL-21-producing CD4^+^ T lymphocytes are also depleted during SIV infection, thus limiting Th17 differentiation pathways, which are controlled by IL-21 *in vivo* (108).

Despite being susceptible to HIV and SIV infections, intestinal Tregs are increased during chronic infections, leading to a decreased Th17/Treg ratio (60, 62, 109). This could be due to limited productive infection and reduced cell death in the Tregs, as well as to an increased differentiation of naïve CD4^+^ T cells into Tregs in the GI tract (109–112).

Note that, in addition to CD4^+^ T-cell loss, RMs infected with SIVmac were reported to suffer a massive loss of the “double positive” (CD4^+^ CD8^+^) T cells, which express high levels of CCR5 and are highly activated (113), within days following SIV infection (30, 62).

### Other Organs

CD4^+^ T-cell depletion is not limited to the GI tract, lymph nodes, or circulation. It also occurs in the spleen and in the liver of NHP, within 21 dpi in the spleen and during the AIDS stage in the liver (67, 114, 115). In the bone marrow, the reduction of the pool of CD4^+^ T cells reflects decreases in circulating CD4^+^ T cells, with the loss affecting mainly memory cells (116). Meanwhile, CD4^+^ T cell counts in the bronchoalveolar lavages (BAL) have been used as a proxy for the CD4^+^ T cell counts in the lung parenchyma. Most CD4^+^ T cells in the lung are of memory phenotype and express CCR5. A nearly complete loss of the CD4^+^ T cells was observed in the BAL by 3 weeks postinfection (114), while other teams reported that both memory CCR5^+^ CD4^+^ T cells and Th17 cell subsets were maintained in the lungs (55, 117). Recently, a study in humanized mice demonstrated that SIV and HIV infections lead to a rapid loss of resident-memory CD4^+^ T cells from the lung interstitium in the first weeks postinfection, which could participate in the increased susceptibility to pulmonary infections (118).

Finally, CD4^+^ T-cell depletion can also be detected in the genital tract. As for CD4^+^ T cells in the GI tract, most of the CD4^+^ T cells found in the vaginal mucosa display an activated, memory phenotype (119). Differently from the intestinal CD4^+^ T cells, almost all CD4^+^ T cells from the vaginal mucosa express CXCR4, while CCR5 is expressed by only half of them. Within 14 dpi, depletion of CD4^+^ T cells, particularly those with CCR5^+^ expression, occurs in the vaginal mucosa of SIV-infected RMs, and lasts throughout the follow-up, until progression to AIDS (119). In HIV-infected women, vaginal CD4^+^ T-cell depletion is strongly correlated to the depletion of circulating CD4^+^ T cells (120). Regarding the male genital tract, CD4^+^ T cells are depleted in the semen of PWH (121) and of SIV-infected cynomolgus macaques during acute and chronic infections (122).

## CD4^+^ T-Cell Dynamics During the Nonpathogenic and Controlled SIV Infections

CD4^+^ T-cell dynamics during nonpathogenic and controlled SIV infections have been extensively studied. Acute SIV infection induces a slight decline in the CD4^+^ T cell counts from the lymph nodes and circulation in natural hosts (e.g., sooty mangabeys, African green monkeys, mandrills etc.), which is followed by a return to virtually preinfection levels at both sites within the first year (47, 123). As a result, the levels of circulating CD4^+^ T cells are virtually normal in chronically SIV-infected African NHPs (124–127). Meanwhile, acute SIV infection induces a massive CD4^+^ T-cell depletion at the mucosal sites, which largely exceeds the number of CCR5-expressing CD4^+^ T cells that is particularly low at the mucosal sites in the natural hosts of SIVs (47, 84). This excess of CD4^+^ T-cell depletion is not due to a different coreceptor usage by SIVs compared to HIV-1, as most SIV use CCR5 (128). The exceptions are strains of SIVsab that naturally infect sabaeus AGMs in West Africa and SIVmnd-1 that infects mandrills, which were reported to also use CXCR4 and/or CXCR6 (129–131), and SIVrcm that naturally infects red-capped mangabeys in West-Central African that was reported to exclusively use CCR2 (132–134). However, *in vivo*, SIVsab was shown to use CCR5 and preferentially deplete CCR5-expressing CD4^+^ T cells (84).

Thus, severe acute CD4^+^ T-cell depletion in GALT is not specific to pathogenic infection, nor is it predictive of the virulence of a retroviral infection, as shown by Pandrea et al., who proposed that the magnitude of the CD4^+^ T-cell restoration was a better predictor of disease progression (84). This conclusion was also supported by studies in rhesus macaques (6). Interestingly, when cross-species infections of rhesus macaques with SIVsmm (47) and SIVagm (48) were performed, they resulted in pathogenic and controlled infections, respectively. Yet, despite these completely opposite outcomes, in both instances, a severe mucosal CD4^+^ T-cell depletion occurred during acute and early chronic infection (47, 48) (**Figure 1**). However, later on in the follow-up, a nearly complete restoration to baseline levels was observed in SIVagm-infected rhesus macaques (48), similarly to long-term nonprogressors (135), while SIVsmm-infected rhesus macaques experienced a progressive loss of intestinal CD4^+^ T cells (47) (**Figure 1**). The SIVsmm-infected rhesus macaques eventually progressed to AIDS (47), and were classified as slow progressors, in comparison to rapid progressors (86, 136) and normal progressors (21, 33). In **Figure 1**, the rapidly progressive and normal progressive SIV infections are illustrated by the dynamics observed in SIVagm-infected pigtailed macaques and SIVmac-infected rhesus macaques, respectively. Note that such different patterns of disease progression can be observed in multiple species.

The presentation of SIV infection in natural hosts is intermediate between the two extreme patterns described above (i.e., pathogenic and controlled infections), consisting of a massive intestinal CD4^+^ T-cell depletion during acute infection followed by a partial CD4^+^ T-cell restoration during chronic SIV infection (**Figure 1**). This pattern is characteristic to SIVsmm infection of sooty mangabeys, SIVagm infection of African green monkeys and patas monkeys, and SIVmnd infection of mandrills (47, 84, 126, 137, 138). A relatively limited impact of the SIV infection on the mucosal CD4^+^ T cells can also be observed in a subset of animals with pathogenic infections, the long-term nonprogressors which restore intestinal CD4^+^ T lymphocytes and CCR5^+^ memory T cells to higher values than normal progressors (97, 139), as long as viral replication is limited (**Figure 1**).

The relatively robust mucosal CD4^+^ T-cell restoration occurs in natural hosts of SIV in the context of the control of chronic T-cell activation and inflammation. Thus, while T-cell activation and inflammation transiently increase during acute SIV infection, immune activation and inflammation are resolved during the transition between acute and chronic SIV infection, in spite of a relatively sustained, robust viral replication (47, 84, 123, 126, 140). This supports a paradigm in which acute CD4^+^ T-cell depletion is driven in natural hosts of SIV by both viral replication and increased inflammation and immune activation, while partial recovery of intestinal CD4^+^ T cells during chronic infection is enabled by the control of immune activation and inflammation, with the remaining mucosal CD4+ T-cell loss being due to the persistent viral replication.

Two important lessons can be drawn from nonpathogenic and controlled SIV infections. First, nonpathogenic SIV infections highlight that a moderate mucosal CD4^+^ T-cell depletion has no discernible pathogenic consequences if immune activation and inflammation are kept at bay. Second, when immune activation, inflammation and viral replication are entirely contained, such as in the controlled SIV infections, total recovery of intestinal CD4^+^ T cells is achievable, although it might take years to reach the preinfection levels (48) (**Figure 1**).

In the natural hosts, the control of the deleterious consequences of SIV infection (which include a moderate chronic CD4^+^ T-cell depletion) resulted from multiple host adaptations that occurred over millions of years of host coevolution with their species-specific viruses (141). One of the keys to this exquisite control of the deleterious consequences of SIV infection in natural hosts of SIVs is the maintenance of the epithelial gut integrity *via* enhanced repair mechanisms (142, 143) and the absence of consequent microbial translocation, which is the main trigger of chronic T-cell activation in pathogenic infections (144). Some of the other host adaptations to elude SIV pathogenicity involve protection from CD4^+^ T-cell depletion, either by preserving the pool of precursors, or by limiting the number of target cells. Interestingly, species which are natural SIV hosts usually present a reduced expression of CCR5 on circulating and mucosal CD4^+^ T cells (41). It has also been reported that Tcm from sooty mangabeys were less frequently infected, potentially due to their lower CCR5 expression (52). By sparing Tcm precursors, as well as Tscm, sooty mangabeys might preserve their capacity to restore the pool of intestinal CD4^+^ T cells (5, 52). Furthermore, lower levels of immune activation and apoptosis of the CD4^+^ T cells from the LNs and circulation may help protect the immune system of the natural SIV hosts from the immune exhaustion described in the late-stage diseases of pathogenic HIV/SIV infections (92, 140, 145). This might be partly due to difference in the dynamics of type 1 interferons. Type 1 interferons are beneficial in the control of SIV infection during acute infection (146, 147), but persistent, dysregulated production is known to contribute to immune activation (147, 148), to induce the expression of proapoptotic markers in uninfected cells (149), and to be associated with disease progression (146). Interestingly, during chronic SIV infection, type 1 interferon response returns to preinfection levels in natural SIV hosts, while it remains elevated during pathogenic infections (62, 150, 151). Thus, the early control of type 1 interferon production in natural SV hosts might also play a role in preventing disease progression, by limiting immune activation and apoptosis of nearby uninfected CD4^+^ T cells.

Additionally, limited CD4^+^ T cell proliferation was described in AGMs, sooty mangabeys, and mandrills, notably among Tcm, with limited to no increase in proliferating CD4^+^ T cells after acute infection (137, 152–156). Additionally, during SIV infection, CCR5 expression is not upregulated on memory CD4^+^ T cells in sooty mangabeys, limiting new rounds of infection (52). By limiting bystander apoptosis, controlling cell proliferation after acute infection, and by limiting upregulation of CCR5 expression on the surface of the CD4^+^ T cells, natural hosts of SIVs limit the production of new susceptible cells which might slow down the pace of CD4^+^ T cell destruction.

Another consequence of the limited expression of CCR5 on the surface of target cells at the mucosal sites is the reduction in the virus ability to initiate mucosal infection (157). Limited expression of CCR5 by the CD4^+^ T cells in the GI mucosa may also significantly impact the rates of maternal-to-infant transmission. CCR5 expression on the CD4^+^ T cells is extremely low at birth and increases with age in both pathogenic and nonpathogenic hosts (156). However, this increase is delayed in natural hosts of SIVs, and memory CD4^+^ T cells from the newborns express lower percentages of CCR5^+^ compared to non-natural SIV hosts, which creates the premise for a reduced rate of maternal-to-infant transmission rates of SIV in natural hosts (about 5%, compared to 20-25% in HIV-1, prior to antiretroviral therapy) (9, 158).

Another significant particularity of several African NHP species is their ability to downregulate CD4 receptor expression at the surface of their CD4^+^ T cells when they enter in the memory pool, rendering them resistant to SIV infection (137, 159, 160).

Through these mechanisms, natural hosts of SIVs spare specific CD4^+^ T-cell subsets, which could contribute to the control of inflammation and maintenance of gut integrity, despite high viral replication during chronic nonpathogenic infections. Multiple immune cell populations are involved in these processes. As an anti-inflammatory milieu, notably containing TGFβ, is rapidly established, this enhances Treg production, thus preventing the chronic immune activation (87). Furthermore, Th17 cells are spared in both gut and blood of SIVsmm-infected SMs and SIVagm-infected AGMs (55, 62, 104). The Th17/Treg ratio remains stable during SIV infection in natural SIV hosts, while it correlates with disease progression in pathogenic infections (62). Similarly, Th17 cells, as well as β7^hi^ CD4^+^ T cells, are maintained in the blood and in the colon of HIV-1 long-term nonprogressors (135). Moreover, the CD4^neg^ CD8α^dim^ T cells and the CD4^neg^ CD8^neg^ (DN) T cells are able to retain some of the helper T cells functions in the African NHPs that are natural hosts of SIV (34, 160, 161).

## Mechanisms of CD4^+^ T-Cell Depletion

**Figure 2:**
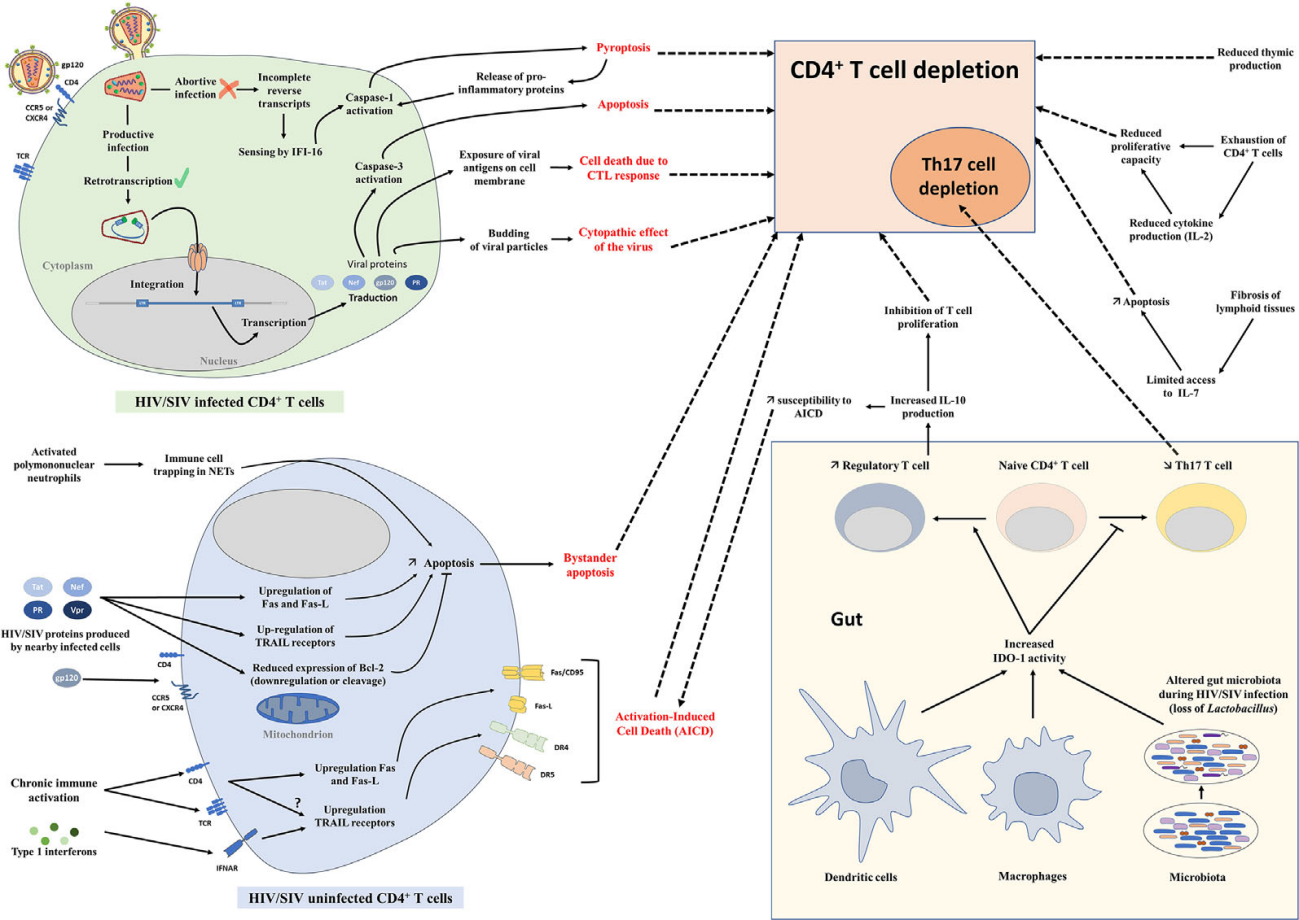
Mechanisms of CD4^+^ T-cell depletion. Schematic representation of different mechanisms involved in CD4^+^ T-cell depletion during HIV and SIV infections. AICD, Activation-induced cell death; CTL, Cytotoxic T lymphocytes; IDO-1, Indoleamine 2,3-dioxygenase 1; NETs, Neutrophil extracellular traps.

The loss of CD4^+^ T cells is caused by different intertwined mechanisms (162). Viral replication significantly contributes at least to the initial CD4^+^ T cell loss, which occurs rapidly in infected individuals and animal models during the acute stage of infection and mirror that of the dynamics of viral replication. Several mechanisms of cell death are directly induced by the infection of those cells by the virus: (i) cytolysis due to increased permeability of cell membrane after viral budding and/or syncytium formation (163), (ii) targeting by HIV/SIV-specific cytotoxic T lymphocytes (164, 165), and (iii) programmed cell death of cells undergoing productive infection, due to caspase-3 and/or Bax activation (166–168) (**Figure 2**). Antibody-dependent or complement-mediated mechanisms are also involved in the destruction of HIV/SIV-infected cells [antibody-dependent cellular cytotoxicity (ADCC) (169), antibody-dependent phagocytosis (170), complement-mediated phagocytosis and lysis (171)], although escape mechanisms have been described for HIV and SIV (172–174).

There are several lines of evidence to support this direct impact of viral replication on CD4^+^ T-cell depletion. First, there is a clear temporal association between viral loads and CD4^+^ T-cell depletion, with the most prominent depletion in the gut closely following the peak of viral replication, which occurs circa one to two weeks postinfection (33, 83, 175) (**Figure 1**). Moreover, there is a clear correlation between the levels of viral replication during acute infection and the magnitude of the CD4^+^ T-cell depletion, particularly in the gut (94). Studies have shown that mucosal depletion is minimal if peak viral loads are below 10^6^ vRNA copies/ml of plasma (48, 137). Furthermore, despite their exquisite ability to finely tune inflammation and T-cell immune activation, NHP species that are natural hosts of SIVs also experience a residual CD4^+^ T-cell depletion during chronic infection when inflammation and immune activation are controlled (47, 84), highlighting the role of viral replication in the persistence of intestinal CD4^+^ T-cell depletion.

Despite this proven impact of viral replication on CD4^+^ T cells at every stage of HIV/SIV infection, the extent of CD4^+^ T-cell loss during the acute infection far exceeds the number of infected lymphocytes (32, 94). Multiple mechanisms have been proposed to explain this excess of CD4^+^ T-cell depletion in HIV infection and pathogenic SIV infections (**Figure 2**): (i) Bystander apoptosis (140, 176), due to viral proteins promoting apoptosis of nearby cells, notably HIV-1 gp120 after its interactions with CD4 and CCR5 or CXCR4 coreceptor (177, 178),; note that in natural hosts of SIVs the levels of bystander apoptosis are kept at bay (84, 140, 145); (ii) Activation-Induced Cell Death (AICD) due to immune activation which induces FasL production and Fas (CD95) expression in nearby, uninfected CD4^+^ T cells, shortening their lifespan and increasing their sensibility to AICD (179–182); interestingly, plasma FasL expression does not significantly increase in animals with nonpathogenic SIV infections (148, 183, 184) (iii) Abortive infection leading to pyroptosis through the caspase-1 pathway, due to an accumulation of incomplete reverse transcripts and induction of antiviral and inflammatory responses (185); (iv) Trapping of immune cells in neutrophil extracellular traps (NETs) induced by SIV infection, followed by an induction of apoptosis or lysis of those trapped CD4^+^ T cells, as recently described (186) (**Figure 2**).

In addition to these general mechanisms involved in total CD4^+^ T-cell depletion, preferential depletion of Th17 cells could be partly due to the induction of indoleamine 2,3-dioxygenase (IDO-1), caused by sustained microbial translocation and immune activation in pathogenic infections (110, 187) (**Figure 2**). Catabolites produced by the degradation of tryptophan by IDO-1 enhance Treg and deplete Th17 cells (110). As adaptive Tregs can produce IL-10 that inhibits T cell proliferation (188) and increases susceptibility to AICD (180, 189), accumulation of Tregs during chronic HIV/SIV infection could also exacerbate CD4^+^ T-cell depletion.

Furthermore, in late stages of HIV/SIV infection, immune exhaustion plays a role in total CD4^+^ T-cell depletion. During chronic infection, increased expression of PD-1 and other immune check-point inhibitors is observed on CD8^+^ T cells, but also on CD4^+^ T cells. Exhausted HIV/SIV-specific CD4^+^ T cells, which are associated with high plasma viremia, have a decreased proliferative capacity and reduced polyfunctional cytokine response, including decreased production of IL-2 (190–192). This fuels gradual CD4^+^ T-cell depletion, in combination with the reduced production of naïve T cells by the thymus (193) and TGF-β-driven fibrosis of lymphoid tissues (194, 195) that are observed during HIV and SIV infections (**Figure 2**).

## Consequences of the CD4^+^ T-Cell Depletion

The consequences of CD4^+^ T-cell depletion have been widely scrutinized, highlighting the critical roles of this cell subset for disease progression and development of comorbidities during pathogenic infections (4, 196–198). The first observation made in patients and NHPs with low peripheral CD4^+^ T-cell counts (<200/mm^3^) was their extreme susceptibility to opportunistic infections (notably fungal infections including *Pneumocystis jirovecii* pneumonia, mycobacterial infections, and cytomegalovirus disease) (199, 200). In addition, PWH with a lower nadir of CD4^+^ T-cell count are also at higher risk of developing AIDS-defining cancers (non-Hodgkin lymphoma, cervical cancer, and Kaposi sarcoma) (201, 202).

However, it was reported that the profound, but transient, CD4^+^ T-cell depletion observed during acute nonpathogenic SIV infections and the residual mucosal CD4+ T-cell depletion persisting during chronic nonpathogenic SIV infections were not sufficient to trigger disease progression (203). As such, a new paradigm emerged in which the combination of CD4^+^ T-cell depletion (notably Th17 cells), inflammation and immune activation in the GI tract drive the deleterious consequences of HIV infection. During HIV/SIV infection, CD4^+^ T cells but also myeloid cells are killed, releasing inflammatory cytokines (204, 205), including IL-1β, thus creating an inflammatory environment (185, 206). Combined with the loss of IL-17 and IL-22-producing cells that are involved in epithelial integrity maintenance and homeostasis, as well as in antimicrobial defense (25, 104, 207), this leads to damage of the gut epithelial integrity, enteropathy and microbial translocation (83, 93, 144). The role of impaired epithelial integrity in driving microbial translocation was confirmed by the demonstration of the leakage of microbial products occurring near breaks in the epithelial lining (144). Microbial translocation can be detected in mucosal tissues (lamina propria, gut-associated lymphoid tissue, mesenteric lymph nodes), but also in distant lymph nodes and circulation (144, 208, 209). These microbial products fuel local and systemic inflammation, and macrophage activation (144, 210). Sustained inflammation and immune activation trigger a vicious cycle by attracting new CD4^+^ T cells, increasing the number of susceptible cells, and by reactivating proviruses in latently-infected cells (206). Newly produced viral proteins and viruses can in turn boost inflammation, tissue damage, and microbial translocation.

The importance of the maintenance of the integrity of the intestinal epithelium was demonstrated recently (209). DSS-induced colitis in SIV-infected AGMs disrupted the intestinal epithelium integrity, recapitulating the characteristics of a pathogenic SIV infection, i.e. increased local inflammation and immune activation, detection of microbial products in lymphoid tissues and increased viral replication (209). Meanwhile, in the inflammatory bowel diseases (IBD), mucosal inflammation is associated with loss of intestinal epithelial integrity and massive infiltration of immune cells, including T cells, in the lamina propria. In response to their exposure to microbial antigens, these T cells produce inflammatory cytokines (IFNγ, TNFα) which disrupt tight-junctions function and worsen intestinal epithelial integrity. However, unlike during HIV/SIV infection, local inflammation does not lead to CD4^+^ T-cell depletion in patients with IBD; on the contrary, most IBD patients present with increased numbers of intestinal CD4^+^ T cells, including Th17 cells (211). As such, comparison with IBD demonstrates that inflammation *per se*, in the absence of the viral trigger, can damage the gut integrity, but it is not sufficient to deplete intestinal CD4^+^ T cells. However, in the context of HIV/SIV infection, inflammation drives T-cell activation (209) and eventually leads to T cell loss through increased viral replication and/or activation-induced cell death (AICD). It is possible that the persistent expression of high levels of type 1 interferons during chronic, pathogenic HIV/SIV infections play a role in this T cell loss, as type 1 interferons are known to induce AICD. Conversely, treatment with type 1 interferons had been evaluated in IBD (212), due to their ability to inhibit Th17 cell differentiation (213).

Chronic inflammation has been linked to numerous non-AIDS comorbidities, notably cardiovascular diseases, liver fibrosis and thromboembolism (214–216). Inflammation and immune activation also promote a procoagulant state in infected animals (217), and they are positively correlated with disease progression (217).

## Restoration of CD4^+^ T Cells During ART

Assessment of the extent of CD4^+^ T-cell restoration in the GI tract that can be expected in patients initiating ART during acute or chronic HIV infection is complex, as most studies focused on the total CD4^+^ T cell counts and only few investigated specific CD4^+^ T-cell subsets, such as memory or Th17 cells. Furthermore, the replenishment of mucosal CD4^+^ T cells can take time, requiring long follow-up of PWH or NHP.

**Figure 3:**
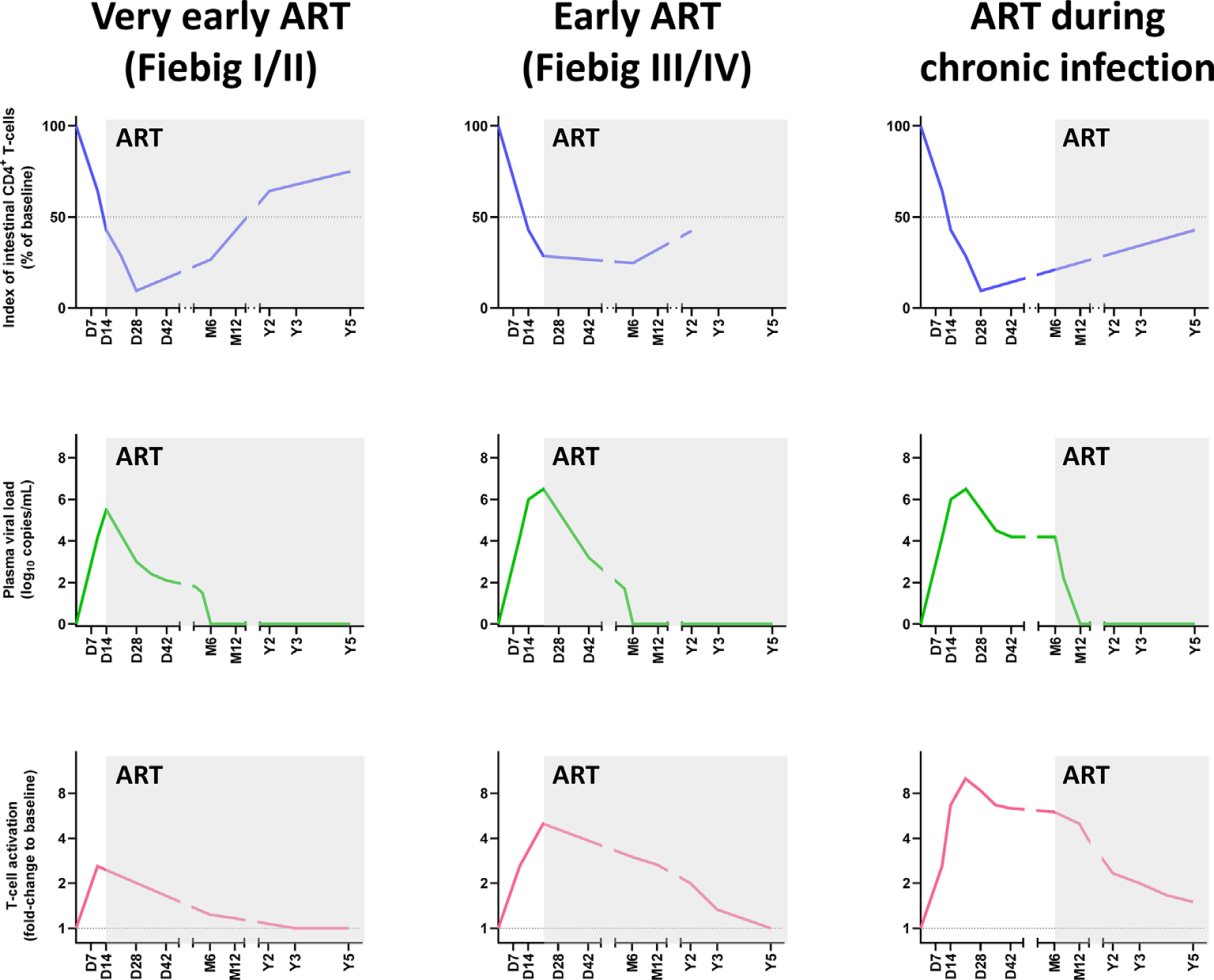
Comparative dynamics of intestinal CD4^+^ T-cell depletion, plasma viral load and immune activation/inflammation in treated HIV infections, according to the timing of initiation of antiretroviral therapy ART. Schematic representation of intestinal CD4^+^ T-cell depletion, viral replication and immune activation is inferred from data reported in references (85, 90, 91, 99, 219–221). Longitudinal data are presented, with the X axis representing days (D), months (M) and years (Y) postinfection. The Y axis illustrates the magnitude of mucosal CD4^+^ T-cell depletion (upper panels), viral replication (middle panels), and the levels of immune activation/inflammation (lower panels). Intestinal CD4^+^ T-cell depletion is illustrated as the index of CD4^+^ T cells (i.e., percentage of CD4^+^ T cell fraction within the CD3^+^ T cell population, divided by this percentage at baseline). Viral replication is represented as plasma viral loads. From the plethora of biomarkers of immune activation/inflammation, we selected the fold-change of HLA-DR^+^ CD38^+^ CD8^+^ T cells in persons living with HIV, compared to uninfected individuals. ART, Antiretroviral therapy.

However, there is a general consensus in the field that the efficacy of the CD4^+^ T-cell restoration on ART vastly depends on the stage of the infection and the degree of immunosuppression at the time of treatment initiation. Guadalupe et al., reported that when ART was initiated at 6 weeks post-HIV infection and was maintained for 14 months, the levels of mucosal CD4^+^ T cells were close to values observed in uninfected individuals (97). Further studies have found that, when ART was initiated in the first weeks postinfection, and viral replication was suppressed in plasma and decreased by 1,000-fold in the GALT, a significant, albeit incomplete, restoration of mucosal CD4^+^ T cells was observed in all humans and macaques (88, 99, 218) (**Figure 3**). In rhesus macaques in which ART was initiated prior to the acute mucosal CD4^+^ T-cell depletion (i.e., 7 days post-SIVmac251 infection), ART failed to prevent CD4^+^ T-cell depletion in the GALT, but enabled a virtually complete CD4^+^ T-cell restoration by 6 months postinfection, particularly through a significant increase in the Tcm levels (88). Meanwhile, while early ART initiation at 3 to 4 days postinfection did not prevent the establishment of the SIV reservoir in lymph nodes (222), it prevented Th17 depletion in the lymphoid tissues (61). Similarly, early treatment of acutely HIV-infected individuals (Fiebig stage I or II) could not halt mucosal CD4^+^ T-cell depletion in the first weeks post-treatment but generated a strong restoration of CD4^+^ T cells in the lamina propria at 96 weeks post-treatment (99) (**Figure 3**).

Meanwhile, most data on patients which initiated ART during chronic HIV/SIV infection suggest a modest CD4^+^ T-cell restoration in the GI tract (97, 98, 219), at least when considering the relative CD4^+^ T cell counts (223). Finally, in patients in which ART was initiated during the AIDS stage, the immune restoration was minimal and occurred very slowly (224) (**Figure 3**).

In patients on ART, a more robust restoration of mucosal CD4^+^ T cells was observed in patients with higher frequency of Tcm in the lamina propria of the jejunum, suggesting that the maintenance and/or the restoration of this subset is critical for an important restoration of intestinal CD4^+^ T cells (219). Furthermore, Th17 cells were also restored in patients receiving ART, especially in those in which therapy was initiated very early in infection (85). However, Th17/Treg ratio remained reduced, as Treg cell counts in lymph nodes and in GALT did not return to baseline levels in PWH receiving ART (60, 61), which might be due to the residual viral replication and immune activation in the GALT of those patients (219).

Overall, as a near-total restoration of mucosal CD4+ T cells is observed only in early ART-treated patients, this is a strong incentive for a generalization of early antiretroviral treatment in all PWH.

## Other Types of CD4^+^ T-Cell Depletion

Important insight on the impact of CD4^+^ T-cell depletion on HIV pathogenesis has been gained by directly depleting the CD4^+^ T cells with monoclonal antibodies, or by using knock-out models in different animal species, as well as through the study of genetic diseases in humans.

### Experimental CD4^+^ T-Cell Depletions

#### Total CD4+ T-Cell Depletion

The first studies on CD4^+^ cell depletion in SIV-infected and SIV-uninfected NHP, performed with anti-CD4 monoclonal antibodies, were published over a decade ago (225–228), and reported that, despite an increased percentage of proliferating (Ki-67^+^) CD4^+^ T cells, reconstitution of CD4^+^ T cell population was slower than what was reported for CD8^+^ T cells in CD8-depleted animals, regardless of SIV infection status (226, 227). Interestingly, CD4^+^ T-cell restoration postexperimental depletion did not differ between natural and non-natural SIV hosts (227), reinforcing the previous finding that the higher restoration of intestinal CD4^+^ T cells in natural hosts of SIV was not due to a higher cell proliferation.

In CD4-depleted, SIV-infected NHPs, plasma viral loads decreased, in relation with the low number of CD4^+^ T cells (225). However, when CD4^+^ T-cell depletion was induced prior to SIV inoculation, this led to persisting high plasma viral loads in CD4^+^ T-cell-depleted monkeys, with no postpeak decline of viremia, and accelerated disease progression (229, 230).

Interestingly, microbial translocation was not increased in SIV-uninfected CD4-depleted animals (227), and CD4^+^ T-cell depletion was not sufficient to reactivate viral replication in CD4-depleted, ART-treated NHPs (228). This limited clinical impact of experimentally-induced CD4^+^ T-cell depletion might be explained by the limited CD4^+^ T-cell depletion at the mucosal effector sites, notably in the GI tract (<50%), in those studies during which anti-CD4 monoclonal antibodies were administered over a short period of time.

#### Selective Treg Depletion

Since Treg are usually accumulating throughout chronic HIV/SIV infection, are frequently infected and suppress HIV/SIV-specific cytotoxic T cell responses (231), different Treg-specific depletion strategies, targeting either CD25 (IL-2 receptor subunit) (232–234) or CCR4 (234, 235), have been investigated. Despite achieving only partial Treg depletion with maximal effect in blood and lymph nodes, and minimal depletion in GI tract, this usually led to increased SIV-specific T cell responses (232, 234), and increased immune activation (232–234). Another strategy aimed at blocking CTLA-4 also resulted in increased SIV-specific T cell responses (236). Viral reactivation occurred in most NHPs in which Treg functions were blocked by anti-CTLA-4 monoclonal antibodies, or in which Treg were depleted (232–234, 237, 238). Higher viral loads in mucosal tissues and greater loss of CCR5^+^ CD4^+^ T cells in the rectal mucosa have been reported to occur in the NHPs receiving an anti-CTLA-4 blocking monoclonal antibody (237).

### Knock-Out Models

Numerous CD4 knock-out mice models have been developed (239–241). In these mice, the TCRαβ^+^ γδ^-^ CD4^neg^ CD8^neg^ (double negative, or DN) T-cell subset is expanded (239). The TCR repertoire of those DN T cells is more polyclonal than in wild-type mice, and these cells are able to maintain part of the helper T cell functions, similarly to natural hosts of SIV (240, 241). However, it was reported that memory cytotoxic CD8^+^ T lymphocytes could be reduced in CD4-deficient mice (242).

### Idiopathic CD4 Lymphopenia

In the late 1980s, a severe lymphopenia, preferentially impacting CD4^+^ T cells, was identified in HIV-uninfected patients with no other condition or treatment known to induce lymphocytopenia (243). This condition was termed idiopathic CD4 lymphopenia (ICL) (244). This disease is rare, with less than 0.5% of blood donors in the United States meeting the definition criteria (245, 246). Due to their low levels of circulating CD4^+^ T cells, ICL patients develop opportunistic infections, some similar to AIDS patients, notably fungal, nontuberculosis mycobacterial and HPV-associated infections (247, 248). A recent work suggests that ICL could have an autoimmune component linked to the production of auto-antibodies directed against CD4^+^ T lymphocytes (249). In some cases, genetic mutations have also been linked to ICL (250).

In addition to CD4^+^ T-cell lymphopenia, an increase in circulating Treg was observed and, in some patients, decreases in CD8^+^ T cells and/or CD19^+^ B cells and/or NK cell counts were also reported (247, 248). Furthermore, CD4^+^ T cells are more activated and proliferating in ICL patients than in controls (247, 251). No specific depletion of CD4^+^ T- cell subsets (Th1, Th2, Th17) was observed in peripheral blood, but a reduction of the percentage of naïve CD4^+^ T cells was seen, compared to controls and PWH (251). Monitoring the CD4^+^ T-cell counts in the mucosal tissues of ICL patients also identified a profound CD4^+^ T-cell loss, although less severe than in PWH, as only a 3-fold reduction in the number of intestinal CD4^+^ T cells was observed (252). This CD4^+^ T-cell loss did not affect the functionality of mucosal Th1 and Th17 cells (252). While an initial study on 10 ICL patients reported a slight increase in microbial translocation (251), a more recent study of 46 ICL patients found normal levels of LPS, and only slight increases of sCD14 (252). We can hypothesize that, similarly to natural hosts of SIV, despite CD4^+^ T cell loss, the maintained functionality of remaining Th17 cells and/or other IL-17 producing cells such as mucosa-associated immune T cells (MAIT) might be sufficient to preserve gut epithelial integrity and limit microbial translocation in ICL patients (253).

### Genetic Mutations

#### Absolute CD4+ T-Cell Depletion

Genetic mutations leading to absolute CD4^+^ T-cell depletion have been reported in two patients, one 22-year-old female with a mutation in the translation initiation codon of the CD4 gene (254) and a 45-year-old female with a mutation in a splice acceptor site leading to the expression of a CD4 protein lacking its anchoring domain to the cellular membrane (255). In the first case, neither CD4 expression on cell membrane, nor soluble CD4 were detected, whereas for the second patient, only CD4 expression on cell membrane was abrogated, while soluble CD4 could still be detected in plasma (254, 255). The first patient was hospitalized for severe viral respiratory infection which led to the discovery of her primary immunodeficiency. Both patients presented numerous HPV-associated warts. However, in both cases, immunodeficiencies were only detected when patients were adults, later than most other primary cellular immunodeficiencies. Interestingly, in both cases, it was shown that helper T cell functions could be performed by DN T cells and/or CD8^+^ T cells (254, 255), similarly to what has been described in in natural hosts of SIV (160, 161) and in CD4 knock-out models in mice (240, 241). The DN T-cell subset was also expanded, similarly to CD4 KO mice. These two reports illustrate that this rescue mechanism can also be found in humans. This absence of CD4^+^ T cells has also been reported in one patas monkey with near-complete loss of peripheral and mucosal CD4^+^ T cells, which protected it from productive SIV infection when intravenously-inoculated with SIVsab (137).

#### Depletion of the Th17 Subset of CD4^+^ T Cells

Patients with hyper-IgE syndromes present with elevated IgE serum levels, decreased Th17 cells, and higher susceptibility to *Staphylococcus aureus* pulmonary, skin infections and *Candida* infections (256, 257). Multiple genetic mutations have been associated with this syndrome, and patients with DOCK8 mutation also present with HPV-associated warts, cutaneous manifestations of *Molluscum contagiosum* and/or Herpes simplex virus infections (258, 259). These studies highlight the importance of Th17 cells in the protection of the organism from bacterial and fungal infections, notably through the maintenance of the integrity of the intestinal barrier, as also emphasized by the increased microbial translocation observed during HIV and SIV pathogenic infections in which Th17 cells are depleted and the intestinal barrier is damaged, with visible breaches in the intestinal epithelium.

## Perspectives for Therapeutic Approaches Aimed at Preventing or Limiting CD4^+^ T-Cell Depletion and Its Consequences

The most effective treatment currently available for preventing or limiting CD4^+^ T-cell depletion is the early initiation of ART, ideally during Fiebig stages I or II. This is the only treatment which has proved a high efficacy in restoring intestinal CD4^+^ T cell in PWH and can have additional positive impact on limiting size of viral reservoirs (**Figure 3**). However, as there is persistent immune activation in PWH on ART, which could cause a limited CD4^+^ T cell loss, early ART might not be sufficient to entirely restore mucosal CD4^+^ T cells in all patients. It should also be acknowledged that, even when viral replication is suppressed, restoration can take time, as evidenced in elite controllers SIVagm-infected RMs, in which complete recovery of intestinal CD4^+^ T cells was only observed after 4 years of absence of viral replication in plasma and tissues (48) (**Figure 1**). Thus, complete recovery of intestinal CD4^+^ T cells in PWH could take even longer, especially if the treatment could not be initiated early in the infection.

As the crucial role of IL-17 and IL-22 producing T cells in preserving the mucosal integrity emerged recently, it has been hypothesized that treatments aiming at maintaining or restoring those cell subsets could limit the deleterious impact of CD4^+^ T-cell depletion in HIV/SIV-infected NHPs, i.e., microbial translocation, inflammation, and immune activation. As pointed out, a long follow-up of PWH receiving those treatments will probably be necessary before being able to definitively rule on their efficacy.

### IL-21

IL-21 has been described to enhance several immune functions, including long-term maintenance of CD8^+^ T cells, differentiation of memory B cells and differentiation of naïve CD4^+^ T cells into Th17 cells (108, 260–262). Several studies have explored its potential to limit Th17 depletion in SIV-infected NHPs. In a preliminary study, Micci et al., observed that, after 5 weekly doses of recombinant IL-21, the frequency of circulating Th17 cells increased in chronically SIVmac-infected macaques (108). Paiardini et al., confirmed these findings in a subsequent study in rhesus macaques treated with IL-21 between weeks 2 and 6 postinfection (263). They observed no difference with respect to the total CD4^+^ T cell counts in circulation, lymph nodes and the GI tract, but intestinal Th17 cells were maintained at week 6 postinfection in IL-21-treated macaques, while a severe depletion was observed in controls (263). This preservation of the Th17 cell subset was associated with lower intestinal inflammation and microbial translocation, as expected (263). Unfortunately, this protective effect on Th17 depletion faded away and intestinal Th17 cell loss was similar in both groups 23 weeks postinfection (263). Similarly, in ART-treated SIV-infected macaques, treatment with IL-21 did not enhance total CD4^+^ T-cell restoration in the circulation, lymph nodes and GI tract, but both intestinal Th17 and IL-22- producing CD4^+^ T cells were restored to near-baseline levels (264). Th17 cells were more frequently polyfunctional in IL-21-treated macaques, and this effect was more robust in jejunum than in rectal biopsies (265). This was sufficient to limit neutrophil infiltration in intestinal tissues, as well as T cell activation and proliferation. However, these positive effects were also blunted over time (264, 265). Conversely, one recent study reported a reduction in immune activation and T-cell exhaustion in IL-21 treated rhesus macaques, but did not see any impact on Th17 CD4^+^ T cells (266).

### IL-7

IL-7 was among the first cytokines investigated, as it was shown to boost CD4^+^ T-cell regeneration (267–269). In SIV-infected rhesus macaques on ART, rsIL-7 induced a transient increase in CD4^+^ and CD8^+^ T-cell counts (270). Similarly, in virologically-suppressed PWH on ART, rhIL-7 increased CD4^+^ T-cell counts in circulation and in the gut (268, 271, 272). Although transient viral reactivations were detected, mainly in patients receiving high rhIL-7 doses (268, 271), and a slight increase in viral reservoir was reported (273), those preliminary results were promising for restoring T cells but clinical trials investigating IL-7 were interrupted due to the appearance of neutralizing antibodies in IL-7-treated patients and production issues.

### IDO-1 Inhibitors

Metabolites generated by the catabolism of tryptophan by IDO-1 (kynurenine pathway) can lead to an increase in the number of Treg while depleting Th17 CD4^+^ T cells (110), Specific IDO-1 inhibitors have been used in oncology, but none has been tested in PWH and only one in SIV-infected RMs (274, 275). Until now, in the HIV/SIV field, in order to reduce IDO-1 expression, most studies focused on altering gut microbiota. A recent study by Vujkovic-Cvijn and colleagues showed that dysbiosis caused by acute SIV infection, notably loss of *Lactobacillus* spp, increased IDO-1 activity and was correlated with Th17 depletion in peripheral blood (187). Interestingly, enhanced IDO-1 activity due to SIV infection could be thwarted by supplementing SIV-infected macaques with *Lactobacillus* (187). The addition of IL-21 did not further lower IDO-1 activity (187, 265). However, the beneficial effect of those probiotic treatments on Th17 cell restoration still has to be demonstrated.

Alterations of intestinal microbiota in PWH and in SIV-infected NHPs have been extensively described (276). In pigtailed macaques (PTM), prebiotics/probiotics improved intestinal CD4^+^ T cell counts, enhanced functionality of colonic Th17 and Th1 CD4^+^ T cells, but did not prevent systemic microbial translocation as shown by the presence of microbial products in peripheral lymph nodes (277). Clinical trials have suggested a potential beneficial effect of probiotics on circulating CD4^+^ T-cell counts or intestinal Th17 cells (278–280). However, the varying compositions of probiotic supplements hindered comparisons between studies, and most of these studies were underpowered due to a low number of included patients. Moreover, other confounding factors complicated the evaluation of those strategies: both HIV-1 and LPS induce IDO-1 expression (112, 281), and thus ART itself could reduce IDO-1 activity in PWH (282).

Another inhibitor of the kynurenine pathway has been recently evaluated in NHPs, a kynurenine 3-monooxygenase inhibitor which increased circulating CD4^+^ T cells but failed to increase intestinal Th17 cell restoration and to prevent microbial translocation (283). Recently, one work reported increased Th17 and Th22 populations among circulating CD4^+^ T cells in ART-treated, SIV-infected rhesus macaques that received a fecal microbial transplantation (284). This restoration of Th17 and Th22 subsets in the blood needs to be confirmed in intestinal tissues in further studies.

### Others

Other strategies have been suggested. One of them consists of targeting CD4^+^ T cells expressing α4β7 integrin, which is a gut-homing signal (285), using an anti-α4β7 monoclonal antibody, to reduce the number of susceptible cells in mucosal tissues and preserve CD4^+^ T cells in the GALT (286). In preliminary works, SIV-infected NHPs receiving anti-α4β7 monoclonal antibody had higher CD4^+^ T cell counts than controls in both peripheral blood and in intestinal tissues (286, 287). However, in PWH, despite a slight increase in the circulating CD4^+^ T cell counts at 10 weeks postinfusion, this increase was not sustained (288). Furthermore, even though gut homing was limited, treatment with anti-α4β7 monoclonal antibody did not prevent HIV or SIV infection, viral reservoir seeding, nor it delayed viral rebound post-treatment interruption (288, 289). Recently, an anti-caspase inhibitor administered to RMs in the first days following SIVmac infection has been shown to reduce T cell death and maintenance of CD4/CD8 T cell ratios (290). Furthermore, memory CD4+ T cells were preserved after the early administration of this inhibitor (290).

## Conclusion

In his literary masterpiece “*The Restaurant at the End of the Universe*”, Douglas Adams states that “It is a curious fact, and one to which no-one knows quite how much importance to attach, that something like 85 percent of all known worlds in the Galaxy, be they primitive or highly advanced, have invented a drink called jynnan tonyx, or gee-N’N-T’N-ix, or jinond-o-nicks, [ … ] ‘chinanto/mnigs,’ [ … ] ‘tzjin-anthony-ks’”. Similarly, acute mucosal CD4^+^ T-cell depletion is a common feature of all HIV and SIV infections, be they pathogenic, nonpathogenic, or controlled. However, as clearly demonstrated by the data presented here, acute CD4^+^ T-cell depletion is only the spark that can ignite the wildfire in the woods, while chronic inflammation and immune activation that lead to comorbidities and disease progression, and the ability of the host to manage these features associated with HIV/SIV infection, are driving the prognosis.

The natural hosts of SIV seem to be also a good example of convergent evolution to develop strategies to thwart retroviral infections. These NHP species are able to constrain this fire to a limited timing by: (i) spacing the trees, i.e. limiting the number of target cells by having a reduced number of CD4^+^ T cells expressing CCR5 and/or down-regulating CCR5 expression when entering the memory pool, (ii) limiting the propagation of fire to unburnt trees, i.e. hampering by-stander apoptosis that is the main driver of cell death in HIV/SIV infections, (iii) preserving specific trees that protects the soil, i.e., Th17 cells that are crucial in the maintenance of gut integrity and protecting from bacterial and fungal infections, or trees that will help the regrowth of the forest, i.e. sparing Tcm cells, that have a higher expansion potential, and (iv) growing fire-resistant trees that are able to maintain wild-life in the absence of the other trees, i.e. CD3^+^ CD4^neg^ CD8^neg^ T cells that exhibit some of the helper T cell functions and that are frequent in most natural hosts of SIV.

## Data Availability Statement

The original contributions presented in the study are included in the article/supplementary material. Further inquiries can be directed to the corresponding author.

## Author Contributions

QLH, IP, and CA designed the manuscript and contributed to drafting. QLH, IS, AL, IP, and CA drafted and revised the manuscript. QLH, IP, and CA prepared the figures. QLH, IS, AL, IP, and CA edited the manuscript. All authors contributed to the article and approved the submitted version.

## Funding

QLH, IP, and CA are supported by grants from the National Institutes of Health/National Institute of Diabetes and Digestive and Kidney Diseases/National Heart, Lung and Blood Institute/National Institute of Allergy and Infectious Diseases: R01DK113919 (IP/CA), R01DK119936 (CA), R01 AI119346 (CA), RO1 HL117715 (IP), R01 HL123096 (IP). The work of IS was supported by the intramural research program of NIAID/NIH. ALL is supported by UM1-AI106701 AIDS Clinical Trials Group Immunology Support Laboratory. The content of this publication does not necessarily reflect the views or policies of the Department of Health and Human Services, nor does mention of trade names, commercial products, or organizations imply endorsement by the U.S. Government. The funders had no role in study design, data collection and analysis, decision to publish, or preparation of the manuscript.

## Conflict of Interest

The authors declare that the research was conducted in the absence of any commercial or financial relationships that could be construed as a potential conflict of interest.
